# Spontaneous calcific cerebral embolization revealing a calcified rheumatic mitral stenosis: a case report

**DOI:** 10.1186/s13256-023-03982-2

**Published:** 2023-06-18

**Authors:** M. Haboub, S. Abouradi, H. Mechal, G. Minko, A. Moukhliss, S. Arous, M. E. G. Benouna, A. Drighil, L. Azzouzi, R. Habbal

**Affiliations:** Cardiology Department, Hospital University Ibn Rochd, Casablanca, Morocco

**Keywords:** Cerebral embolism, Calcified mitral stenosis, Transient ischemic attack

## Abstract

**Background:**

Cerebral cardiac embolism accounts for an increasing proportion of ischemic strokes and transient ischemic attacks. Calcified cerebral emboli are rare and mostly iatrogenic secondary to heart or aorta catheterization. However, spontaneous cerebral calcified embolism in the case of calcified aortic valve is very rare and there are less than 10 case reports in the literature. And a more interesting fact is that such an event, in the context of calcified mitral valve disease, has never been reported, at least to our knowledge. We are reporting a case of spontaneous calcified cerebral embolism revealing a calcified rheumatic mitral valve stenosis.

**Case presentation:**

We report a case of a 59 year-old Moroccan patient, with a history of rheumatic fever at the age of 14 and no history of recent cardiac intervention or aortic/carotid manipulation, who was admitted to the emergency department after a transient ischemic attack. Physical examination at admission found normal blood pressure of 124/79 mmHg and heart rate of 90 bpm. A 12-lead electrocardiogram showed an atrial fibrillation, no other anomalies. Unenhanced cerebral computed tomography imaging was performed, revealing calcified material inside both middle cerebral arteries. Transthoracic echocardiography was performed, showing severe mitral leaflets calcification with a severe mitral stenosis, probably due to rheumatic heart disease. Cervical arteries Duplex was normal. A vitamin K antagonist (acenocoumarol) was prescribed, targeting an international normalized ratio of 2–3 and mitral valve replacement surgery was performed using mechanical prosthesis. Short- and long-term health, with a 1-year follow-up, were good and the patient did not experience any stroke.

**Conclusion:**

Spontaneous calcified cerebral emboli secondary to mitral valve leaflet calcifications is an extremely rare condition. Replacement of the valve is the only option to prevent recurrent emboli and outcomes are still to be determined.

## Background

Cerebral cardiac embolism accounts for an increasing proportion of ischemic strokes and transient ischemic attacks [1]. Calcified cerebral emboli are rarely reported, but potentially cause of strokes and transient ischemic attacks and may be the first manifestation of vascular or cardiac disease. Identification of the source of embolization is crucial to prevent future emboli, neurological damage, and death. Non-contrast computed tomography (CT) scan of the head is the most common imaging procedure used as the initial assessment of suspected stroke or transient ischemic attack [2].

Cerebral calcified embolus can occur after percutaneous and surgical intervention in the context of calcified aortic or mitral valve disease [3]. These emboli are presumed to occur because of valve trauma. However, spontaneous cerebral calcified embolism in the case of calcified aortic valve is very rare and there are less than ten case reports in the literature [4]. And a more interesting fact is that such an event in the context of calcified mitral valve disease has never been reported, at least to our knowledge.

We are reporting a case of spontaneous calcified cerebral embolism revealing a calcified rheumatic mitral valve stenosis.

## Case presentation

**Fig. 1:**
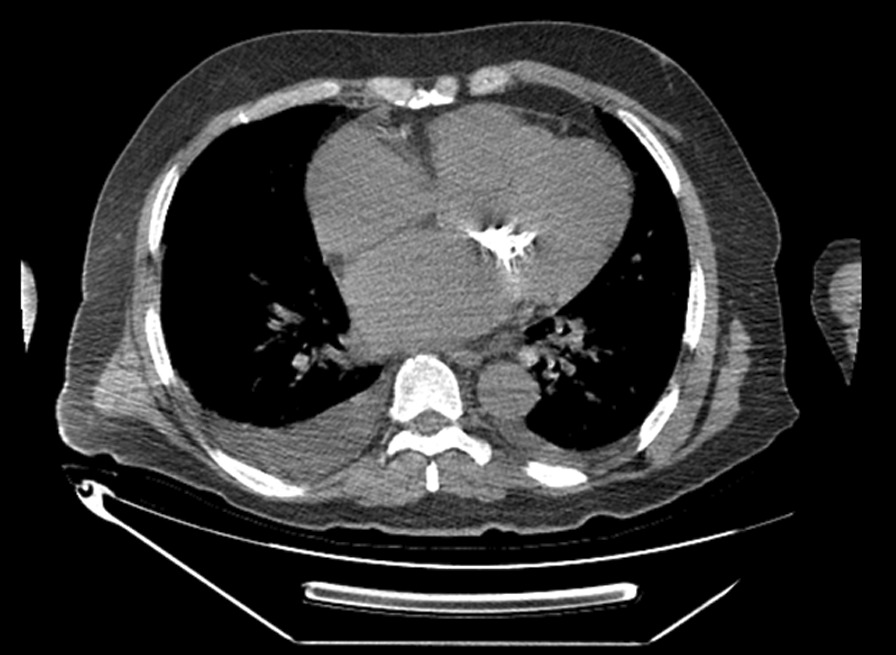
Chest computed tomography confirming mitral leaflet calcification

We are reporting a case of a 59-year-old Moroccan man presenting to the emergency department after a transient ischemic attack (right hemiparesia and left central facial paralysis resolving briefly and spontaneously). There was a history of rheumatic fever at age 14 and stage II New York Heart Association (NYHA) dyspnea on moderate exertion for 2 years, with no history of recent cardiac intervention or aortic/carotid manipulation and no other symptoms. Physical examination on admission found an irregular heart rhythm of 90 bpm, blood pressure (BP) of 124/79 mmHg, mid-diastolic rumble at apex, no signs of heart congestion, and no signs of neurological impairment. A 12-lead-electrocardiogram showed an atrial fibrillation without other anomalies. Two-dimensional (2D) transthoracic echocardiograms revealed important mitral valve leaflets calcifications, probably related to rheumatic heart disease; planimetry of the valve was not possible. Continuous wave Doppler interrogation of the mitral valve found a severe mitral stenosis with a mean gradient of 15 mmHg and a continuity equation surface of 1 cm^2^. The aortic valve was thickened, but not calcified, a moderate aortic regurgitation was noticed. Left ventricular (LV) function was normal and the LV ejection fraction (LVEF) was at 55%. There was a right ventricular (RV) longitudinal systolic dysfunction: TAPSE 11 mm and S’VD 6 cm/second. Tricuspid valve was thin with a mild tricuspid regurgitation. Continuous wave Doppler interrogation of the tricuspid valve allowed to estimate systolic pulmonary artery pressure at 69 mmHg. Mitral valve calcification was best shown on a cardiac CT (Fig. 1).

**Fig. 2:**
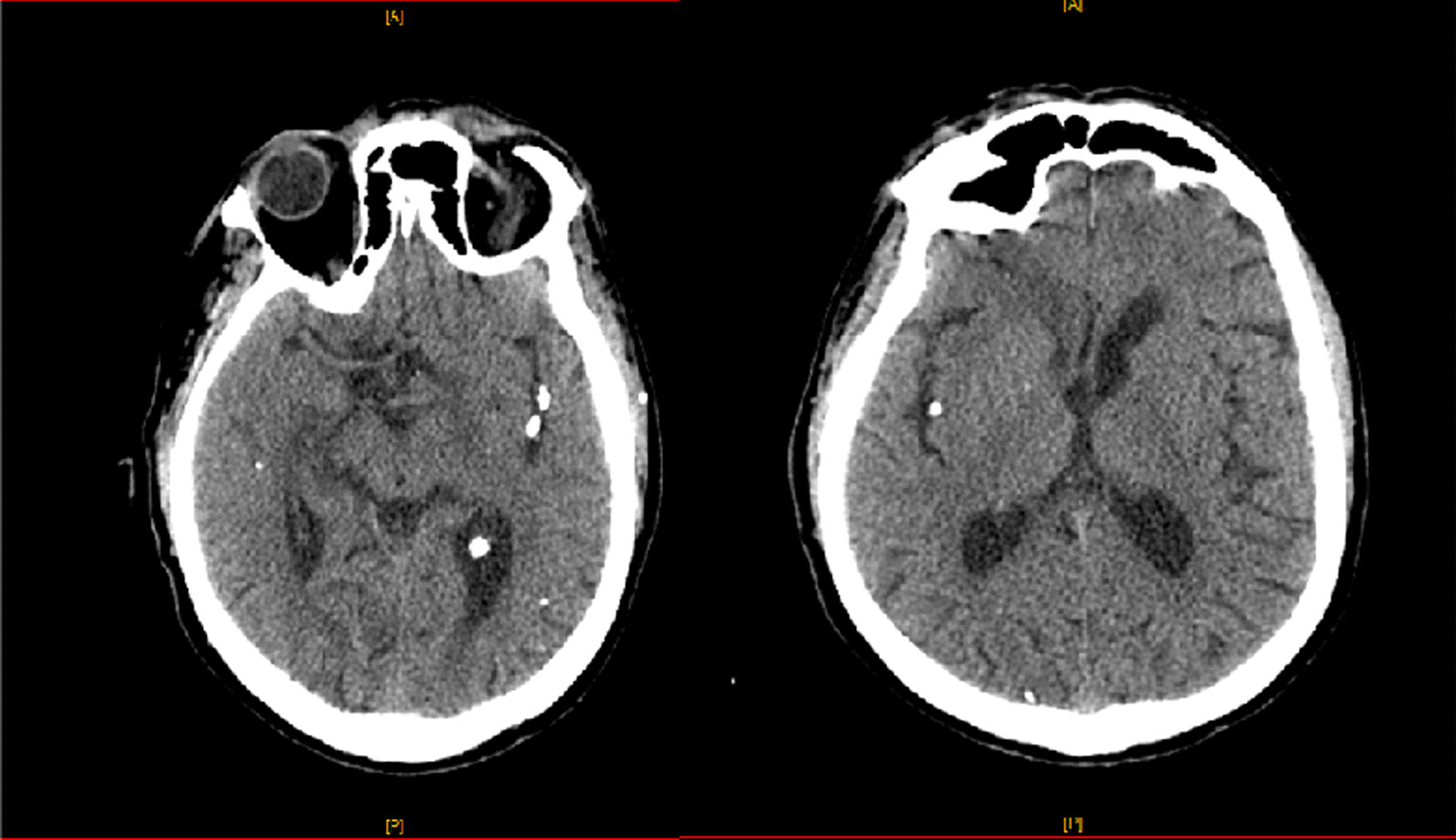
Unenhanced cerebral computed tomography with axial reconstructions showing calcified emboli in both middle cerebral arteries. These arteries were permeable

**Fig. 3:**
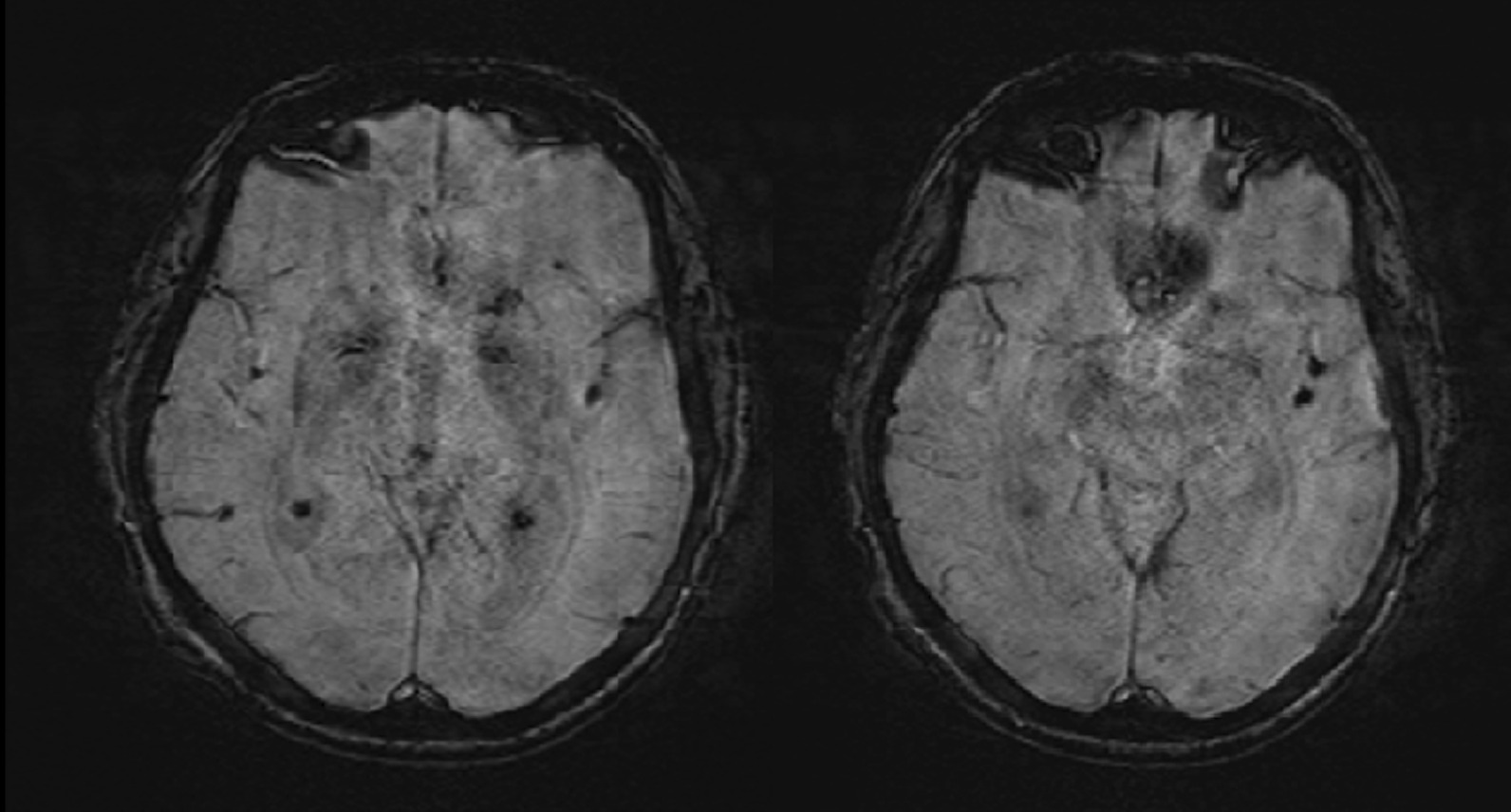
Susceptibility weighted cerebral magnetic resonance sequences showing absence of signal inside both middle cerebral arteries

Unenhanced cerebral CT was performed, revealing calcified emboli in both middle cerebral arteries (M3 and M4 segments) (Fig. 2). Susceptibility weighted magnetic resonance cerebral sequences result is reported in Fig. 3.

Cervical arteries Duplex showed normal carotid and vertebral arteries.

A vitamin K antagonist (acenocoumarol) was prescribed, targeting an international normalized ratio (INR) of 2–3 and replacement of mitral valve using a mechanical prosthesis was performed with a good short-term and long-term outcome. The patient did not experience ischemic stroke during a 1-year follow-up.

## Discussion

In our patient, transient ischemic attack (TIA) was certainly due to atrial fibrillation (AF), and cerebral imaging lead to the discovery of calcified emboli.

Approximately 6%–31% of TIA are caused by a cardiogenic cerebral embolism (cardioembolic TIA) [5, 6]. Determining TIA etiology is important before administering therapy. Permanent or paroxysmal, valvular and non-valvular, AF is associated with a three- to five-fold increased risk of TIA and stroke [7, 8]. Cardiogenic cerebral embolization is common among patients with any cause of AF, but particularly in AF resulting from rheumatic and arteriosclerotic heart disease [9]. It is recommended to prescribe these patients oral anticoagulant therapy in case of valvular AF and, according to CHA2DS2VASc score, in case of non-valvular AF.

Calcified cerebral emboli are an infrequent, but increasingly recognized cause of TIA and ischemic stroke, although recognition among general radiologists and clinicians can be limited. Unenhanced CT and computed tomography angiography (CTA) are the imaging techniques of choice for the diagnosis [10, 11]. First described on CT by Yock in 1981, calcified cerebral emboli were previously thought to be unusual, and to most commonly arise following instrumentation of calcified cardiac valves or direct aortic/carotid artery manipulation [12, 13]. However, there is growing evidence that spontaneous calcified cerebral embolism is more common, with a recent study and review of published cases reporting a 2.7% prevalence among a group of patients presenting with suspected stroke over a 1-year period. In this report, the middle cerebral artery was the site of 83% calcified emboli. Cardiac valvular disease was more common than carotid atheromatous disease, with calcified aortic stenosis three times more common than mitral valve disease as the embolic source [3].

Iatrogenic calcified embolus following cardiac surgery or catheterization is common [4, 14, 15]. These emboli are presumed to occur because of valve trauma. According to a most recent postmortem analysis of iatrogenic embolization cases, the source of calcified cerebral emboli was attributed to dislodgement and displacement of calcified material from calcified aortic valves and ulcerated aortic atherosclerotic plaques during therapeutic and investigative procedures [16].

There are some reported cases of cerebral calcific emboli following open heart mitral valvotomy and percutaneous mitral valvuloplasty. Mitral calcification accounts for fewer than 1% of cerebral cardiac embolism, and in all described cases, it was secondary to mitral valve intervention [16]. However, there is not any case report published describing spontaneous calcified cerebral emboli in the context of calcified rheumatic mitral stenosis.

In case of stroke secondary to calcified emboli, the role of thrombolysis remains uncertain, as there are conflicting reports regarding its efficacy in this setting [17–19]. There is debate and very limited experience regarding the place of mechanical thrombectomy [18, 20]. Subsequent imaging evaluation of this subgroup of patients who have suffered from ischemic strokes requires caution because the calcified nature of the embolus may be obscured on CT angiography or magnetic resonance imaging (MRI). Clinical evaluation should include consideration of potential proximal source of calcified material and recent cardiac intervention or aortic/carotid manipulation. Although there is no data showing benefit of valve replacement, most authors advocate valve replacement to remove the source of emboli.

## Conclusion

Spontaneous calcified cerebral emboli, secondary to mitral valve leaflet calcification is an extremely rare condition. Replacement of the valve is the only option to prevent recurrent emboli and outcomes are still to be determined.

## Data Availability

The published information is available from the corresponding author on reasonable request.
